# One Major Challenge of Sequencing Large Plant Genomes Is to Know How Big They Really Are

**DOI:** 10.3390/ijms19113554

**Published:** 2018-11-11

**Authors:** Jaroslav Doležel, Jana Čížková, Hana Šimková, Jan Bartoš

**Affiliations:** Institute of Experimental Botany, Centre of the Region Haná for Biotechnological and Agricultural Research, Šlechtitelů 31, CZ-78371 Olomouc, Czech Republic; cizkova@ueb.cas.cz (J.Č.); simkovah@ueb.cas.cz (H.Š); bartos@ueb.cas.cz (J.B.)

**Keywords:** flow cytometry, genome size, nuclear DNA content, reference genome assembly, standardization

## Abstract

Any project seeking to deliver a plant or animal reference genome sequence must address the question as to the completeness of the assembly. Given the complexity introduced particularly by the presence of sequence redundancy, a problem which is especially acute in polyploid genomes, this question is not an easy one to answer. One approach is to use the sequence data, along with the appropriate computational tools, the other is to compare the estimate of genome size with an experimentally measured mass of nuclear DNA. The latter requires a reference standard in order to provide a robust relationship between the two independent measurements of genome size. Here, the proposal is to choose the human male leucocyte genome for this standard: its 1C DNA amount (the amount of DNA contained within unreplicated haploid chromosome set) of 3.50 pg is equivalent to a genome length of 3.423 Gbp, a size which is just 5% longer than predicted by the most current human genome assembly. Adopting this standard, this paper assesses the completeness of the reference genome assemblies of the leading cereal crops species wheat, barley and rye.

## 1. Introduction

The more that is known regarding the organization and function of plant and animal genomes, the more it becomes clear that a full understanding of genome function will require the acquisition of a complete sequence. The enormous throughput offered by current short read DNA sequencing technologies allows for the sequencing of genomes of any size and at a high sequencing depth. While this enables the ready assembly of single and low-copy sequences, the inclusion within the assembly of repetitive sequence is a non-trivial challenge, and, together with sequence redundancy due to polyploidy, represent a major obstacle to the acquisition of gap-free long-range genome sequences.

A reference genome assembly aims to faithfully represent a complete genome sequence, ideally with each chromosome being represented by a single, gap-less pseudomolecule. The level of completeness of an assembly remains difficult to ascertain, however, especially in the case of complex genomes, in which tracts of repetitive DNA, segmental duplications and, in the case of polyploid genomes, the presence of homoeologs, are all inimical to the elaboration of a “correct” assembly: the result is that gaps, mis-assemblies and collapsed tandem repeats feature in most published genome sequences. A much-used computational method to size a nuclear genome relies on the concept of k-mer frequencies [1,2]. An alternative may be to determine the number of full-length LTR-retrotransposons. As their number increases linearly with genome size, at least in grass species, it may serve as a measure of assembly quality [3]. Genome size of unknown species might then be obtained by extrapolation, using data from species whose genome size is known. However, as both approaches rely on sequence data, the only truly independent way to determine genome size is to experimentally determine the quantity of DNA present in the nuclei.

## 2. Estimation of Genome Size

Two experimental approaches have been developed to estimate nuclear DNA amounts: biochemical and cytometric. The former seeks to quantify the DNA harbored within a known mass of plant tissue [4]; its weakness lies in the errors inherent in the estimation of the number of nuclei present in the sample, in the unknown proportion of nuclei present at each of the various different cell cycle stages and the non-estimable proportion of endo-reduplicated nuclei present. As a result, cytometry-based estimations tend to be preferred, since these are designed to quantify the DNA present in a population of nuclei at a known cell cycle stage [5]. The attempt by [6] to derive relative nuclear DNA amounts present in several plant species using Feulgen micro-densitometry led to the development of the now universally understood C-value terminology, where un-replicated haploid nuclei contain a 1C DNA amount; the terminology has been refined in recent years [7]. Feulgen microdensitometry was phased out during the 1980s as a result of the throughput benefits offered by flow cytometry, which offers the possibility of analyzing large numbers of isolated nuclei in a short time [5].

It is important to note that flow cytometry does not quantify nuclear DNA directly, but rather achieves this by capturing the signal emitted from fluorochrome-stained nuclei. In order to determine a nuclear DNA amount in absolute units, the fluorescence of an unknown sample has to be compared with that of a reference standard of known genome size [8]. To avoid errors due to non-linearity, an ideal reference standard should not differ in size by more than two or three-fold from the test sample, implying that a set of reference standards is needed in order to cope with the large range of genome size encountered among higher organisms. The question then becomes how to calibrate these reference standards if none of the candidate species has itself been completely sequenced.

## 3. Standardization

Not unexpectedly, the issue applies as much to animal to plant or fungal genomes. To enable a comparison of data obtained by different laboratories, Tiersch et al. [9] calibrated a set of animal reference standards, choosing human male leukocytes (7 pg DNA/2C) as the primary reference; the 7 pg figure was based on estimates derived from Feulgen micro-densitometry [10]. The experiments derived a 2C value of 2.5 pg DNA for domestic chicken, which was close to the value given by [11]. The domestic chicken genome has been adopted since this time as the most widely used reference standard for the sizing of animal genomes [12]. In an effort to enable comparisons between animal and plant genomes, Doležel et al. [8] recommended a set of plant reference standards (Table 1), also calibrated with respect to the human male leukocyte genome, assuming the 7 pg value assigned by Tiersch et al. [9]. Over the past three decades, hundreds of genome size estimates have been published, based mainly on the 7 pg value. The question is how close to reality these estimates really are, which relates in the main to how accurate the 7 pg figure is. According to the arguments made by Doležel and Greilhuber [13], the value most probably over-estimates the true value by 5–10%.

## 4. The Human Genome as a Universal Reference Standard

Seventeen years have passed since the joint announcement of the human genome sequence [14,15]. This period has seen a number of attempts to complete the assembly, applying a variety of technologies [16,17]. All of these have reported a smaller genome size than what has, as of the end of 2017, been suggested in GRCh38.p12, the most recently released Genome Reference Consortium version, which comprises 3,257,319,537 bp. Assuming the Doležel et al. [18] conversion of 1 pg = 0.978 Gbp, 3.5 pg 1C DNA is equivalent to 3,423,000,000 bases. Thus, the 7 pg value represents an ~5.1% over-estimate of the GRCh38.p12 assembly prediction. This difference lies at the lower end of the error range predicted by Doležel and Greilhuber [13]. Given that the human reference genome is still incomplete, the expectation is that the gap between the 7 pg figure and the “real” human genome size will continue to diminish. Nevertheless, a 5% error is not dissimilar to the variation observed between estimates of nuclear DNA amounts of a given species produced by different laboratories [19,20]. Thus, the recommendation remains that the 7 pg figure continue to be used as the reference for measuring 2C values of both animal and plant genomes.

## 5. Sizing the Large Triticeae Genomes

Three species belonging to the tribe Triticeae—namely, bread wheat (*Triticum aestivum*), barley (*Hordeum vulgare*) and to a lesser extent, cereal rye (*Secale cereale*)—provide a major proportion of the calories used by humans and their livestock across the temperate world. The acquisition of their genome sequences will facilitate marker- and genomics-assisted breeding, gene editing and other novel breeding technologies currently under development. Reference genome sequences have been published for barley [21], wild emmer wheat (*T. dicoccoides*) [22] and hexaploid bread wheat (*T. aestivum*) [3], and one for cereal rye is currently being finalized (Nils Stein, pers. comm.). Here, flow cytometry was utilized to assess the nuclear DNA content of wild emmer, bread wheat, barley and cereal rye. To minimize errors due to copy number variants and intraspecific differences in genome size, the accessions of each species were those used for the acquisition of their genome sequences. The cereal rye cultivar Daňkovské (16.19 pg/2C) and garden pea (*Pisum sativum*) cultivar Ctirad (9.09 pg/2C) were used as reference standards (Table 1). Rye was selected out of the calibrated reference standards (Table 1) as its 2C value was close to 2 C DNA amounts of tetraploid and hexaploid wheat and barley. However, this standard could not be used for another accession of rye and thus pea was employed as the second standard. The outcomes are summarized in Table 2.

## 6. Completeness of the Current Triticeae Reference Genome Assemblies

To estimate the completeness of reference genome assemblies of the four test-species, the sizes predicted by each of their assemblies were compared with their estimated genome sizes as derived by flow cytometry. Taking the [9] figure of 7 pg DNA/2C, the conclusion was that the Triticeae assemblies represent at least 85% of their full genome (Table 3). However, adopting the GRCh38.p12 with 1C genome size of 3,257,319,537 bases as the reference, increased the coverage to at least 88%. It should be noted, however, that the data on the size of genome assembly do not inform about its quality, i.e., the correct ordering and orientation DNA contigs. This parameter needs to be assessed using other methods than flow cytometry.

## 7. Concluding Remarks and Recommendations

Cytometric methods suitable for the estimation of nuclear genome size independent of DNA sequence data require a reference standard of known genome size. The most widely used animal and plant DNA reference standards have been calibrated from the human male leucocyte genome, assuming its length to be 3.42 Gbp/1C (and its 2C content to be 7 pg DNA), even though the length estimate is 5.1% greater than what the most current assembly predicts; however, given that the GRCh38.p12 assembly is most probably still incomplete, the real difference may be smaller than this. Thus, for the moment, it would seem reasonable to continue with this figure. The use of an agreed standard will facilitate comparisons between results obtained in different laboratories. Once the human genome size is known to a yet higher level of precision, it will be straightforward to recalculate the size of genomes estimated to date.

## 8. Materials and Methods

Grain of hexaploid bread wheat cultivar (cv.) Chinese Spring were obtained from *P. Sourdille* (INRA Clermont-Ferrand, Clermont-Ferrand, France), those of *T. dicoccoides* (accession Zavitan) from A. Distelfeld (Tel Aviv University, Tel Aviv, Israel), those of barley cv. Morex from Nils Stein (IPK, Gatersleben, Germany) and those of cereal rye inbred line Lo7 from Eva Bauer (Technische Universität Munich, Munich, Germany). Grains of cereal rye cv. Daňkovské and seed of pea cv. Ctirad were obtained from, respectively, the Oseva Agro (Brno, Czech Republic) and Semo (Smržice, Czech Republic) breeding stations. Plants were raised in garden compost in pots and maintained in a greenhouse until they reached a height of 10–15 cm. Nuclei were extracted from leaves and suspended in preparation for flow cytometry following the methods given by [23]. Briefly, 10 mg of leaf tissue of each of the sample species and one of the two reference standards were chopped together in a 1 mL volume of LB01 solution [23] using a razor blade. The resulting homogenate was filtered through a 50-µm nylon mesh. The filtrate was made up to 50 µg/mL RNase and 50 µg/mL propidium iodide, and subjected to flow cytometry using a CyFlow Space flow cytometer (Sysmex Partec GmbH, Görlitz, Germany) equipped with a 532 nm green laser. The gain of the instrument was adjusted so that the peak representing G1 nuclei of the standard was positioned approximately on channel 100 on a histogram of relative fluorescence intensity when using a 512-channel scale. Five individual plants per each test species were sampled, and each sample was analyzed three times, each time on a different day. A minimum of 5000 nuclei per sample was analyzed and 2C DNA contents (in pg) were calculated from the means of the G1 peak positions by applying the formula (sample G1 peak mean) × (standard 2C DNA content)/(standard G1 peak mean). DNA contents in pg were converted to genome lengths in bp using the factor suggested by Doležel et al. [18], i.e., 1 pg DNA = 0.978 Gbp.

## Figures and Tables

**Table 1:** Plant DNA reference standards calibrated for the estimation of nuclear DNA amounts in absolute units [8].

Plant Species and Cultivar *	2C DNA Content (pg DNA) **
*Raphanus sativus* L. ‘Saxa’	1.11
*Solanum lycopersicum* L. ‘Stupické polní rané’	1.96
*Glycine max* Merr. ‘Polanka’	2.50
*Zea mays* L. ‘CE-777’	5.43
*Pisum sativum* L. ‘Ctirad’	9.09
*Secale cereale* L. ‘Daňkovské’	16.19
*Vicia faba* L. ‘Inovec’	26.90
*Allium cepa* L. ‘Alice’	34.89

* Seeds of the reference standards can be obtained from the corresponding author free of charge at dolezel@ueb.cas.cz. Since the year 2000, seed samples were provided to 615 research projects worldwide. ** Estimated after considering 7 pg DNA/2C for human [9].

**Table 2:** Estimation of nuclear DNA amounts in the four Triticeae species.

Species and Genotype	2C Nuclear DNA Content (pg) *		Reference Standard
	Mean	± SD	
*Triticum aestivum* cv. Chinese Spring	33.91	0.27	*Secale cereale* cv. Daňkovské
*Triticum dicoccoides* cv. Zavitan	25.11	0.16	*Secale cereale* cv. Daňkovské
*Hordeum vulgare* cv. Morex	10.31	0.09	*Secale cereale* cv. Daňkovské
*Secale cereale* inbred line Lo7	15.95	0.11	*Pisum sativum* cv. Ctirad

* Considering 7 pg DNA/2C for human [9].

**Table 3:** The estimated level of completeness of the four Triticeae reference genome assemblies.

Species	Reference Genome Assembly (Gbp) *	Flow Cytometric Estimation of 1C Genome Size **			
		GRCh38.12		[9]	
		Genome Size (Gbp)	Assembly Coverage (%)	Genome size (Gbp)	Assembly Coverage (%)
*H. vulgare*	4.79	4.88	98	5.04	95
*S. cereale*	6.67	7.42	90	7.80	86
*T. dicoccoides*	10.50	11.87	88	12.28	85
*T. aestivum*	14.50	16.03	90 ***	16.58	87

* Reference genome assemblies: *H. vulgare* [21], *T. dicoccoides* [22], *T. aestivum* [3], *S. cereale* (Nils Stein, pers. comm.). ** Two different values were used for human 1C genome size as a primary reference standard: 3,257,319,537 bp (GRCh38.p12) and 3,423,000,000 bp [9]. *** Slightly higher value (92%) was estimated by the International Wheat Genome Sequencing Consortium [3] when considering human genome size of 3,253,848,404 bases (Human Genome Assembly GRCh38.p11).
